# Novel technologies to characterize and engineer the microbiome in inflammatory bowel disease

**DOI:** 10.1080/19490976.2022.2107866

**Published:** 2022-09-14

**Authors:** Alba Boix-Amorós, Hilary Monaco, Elisa Sambataro, Jose C. Clemente

**Affiliations:** a Department of Genetics and Genomic Sciences, Precision Immunology Institute, Icahn School of Medicine at Mount Sinai. New York, NY, USA; b Department of Biological Sciences, CUNY Hunter College, New York, NY, USA

**Keywords:** microbiome, IBD, technology, flow cytometry, metabolomics, networks, time series, microbial therapeutics

## Abstract

We present an overview of recent experimental and computational advances in technology used to characterize the microbiome, with a focus on how these developments improve our understanding of inflammatory bowel disease (IBD). Specifically, we present studies that make use of flow cytometry and metabolomics assays to provide a functional characterization of microbial communities. We also describe computational methods for strain-level resolution, temporal series, mycobiome and virome data, co-occurrence networks, and compositional data analysis. In addition, we review novel techniques to therapeutically manipulate the microbiome in IBD. We discuss the benefits and drawbacks of these technologies to increase awareness of specific biases, and to facilitate a more rigorous interpretation of results and their potential clinical application. Finally, we present future lines of research to better characterize the relation between microbial communities and IBD pathogenesis and progression.

## Introduction

Developments in microscopy by van Leeuwenhoek revolutionized our understanding of microbes. Similarly, technological advances, such as high-throughput sequencing, mass spectrometry, and computational tools for data analysis have tremendously contributed to the characterization of microbial communities in human health and disease. Specifically, it is now widely accepted that microbes play a fundamental role in the pathogenesis, disease progression, and response to treatment in inflammatory bowel disease (IBD). IBD is a devastating condition characterized by chronic inflammation of the digestive tract and for which there is currently no cure. While genetics is a determining factor in establishing disease risk,^1,2^ environmental factors, such as the microbiome, are thought to contribute as triggering elements that can modulate the natural history of the disease.^3–5^ Understanding the interaction of host and microbes is therefore required for a more complete characterization of IBD and to potentially identify therapeutic targets.

Here, we review recent technological advances of relevance that are being utilized to understand how the host and the microbiome interact in the context of IBD (Table 1). We review experimental technologies that provide more accurate identification of microbes, describe methods to uncover microbial products responsible for pathogenic mechanisms, present novel techniques for data analysis, and discuss state-of-the-art approaches that aim at modifying the microbiome for therapeutic purposes. We further describe the limitations of these methods and discuss opportunities for future avenues of research.

**Table 1.** Tools for characterizing the IBD Microbiome.

Technology	Tool description	Relevance to IBD	Drawbacks
**Experimental Methods**			
IgA-Seq	Flow cytometry method that allows bacteria coated with IgA to be separated from bulk and identified by sequencing methods.^6–10^	Identified increased levels of IgA-coating in IBD and differential binding of pathobionts^7,11,12^ i.e. invasive *E.coli* in CD patients.^13^ Disrupted IgA recognition of pathogenic morphologies of *C.albicans*.^14^	This technique captures immune-recognized microbes, but discrimination of pathobionts from commensals can be challenging.
GC/MS and LC/MS	Mass spectrometry methods facilitating the characterization of the metabolic environment in the gut.	Identification of metabolomic biomarkers for IBD.^5,15^ Identified lower levels of short-chain fatty acids,^16–19^ tryptophan,^20–22^ and bile acid^23,24^ metabolism in IBD.	Sample preparation influences which metabolites are extracted, ionized and detected.^25^ Instrument differences can impact molecular resolution, and method of choice (targeted vs untargeted) affects specificity, sensitivity and quantification.^26^
SCFA Derivatization	SCFAs are small and volatile, which makes identification difficult with traditional GC/MS approaches. New derivatization techniques have recently been developed to improve SCFA quantification.^27–32^	SCFAs play a role in numerous cellular and immunological processes, such as stimulating the production of mucins, reducing intestinal permeability and promoting anti-inflammatory pathways.^33^ Restoration of SCFA via direct administration or restoration of their microbial producers are being explored as therapeutics for IBD.^34–36^	Time-consuming, and can affect simultaneous measurement of SCFAs and other molecules in the samples.^28^
Ultrahigh-Performance metabolomics to detect low abundant Trp metabolites	Liquid chromatography tandem mass spectrometry with electrospray ionization (UHPLC-ESI-MS/MS) was recently designed to study Trp metabolites. Offers better resolution and allows measurement of low-abundance metabolites downstream of Trp metabolism.^37^	Some common metabolomic methods focus on major metabolites, but other less-abundant downstream-metabolic products are also altered in IBD.^20,38^	High cost. Some metabolites may be unstable in solution. May need optimization to increase confidence in the concentration of certain metabolites.^37^
**Computational Methods**			
Strain-level identification of bacteria	MetaPhlAn2/3,^39,40^ Kraken2,^41^ Strainer,^42^ StrainFinder,^43^ mOTUs,^44^ inStrain.^45^	MetaPhlAn2 has been applied to IBD cohort data to identify and define IBD-like consortia^15,46–48^ and predict treatment response.^49^	Data processing by different metagenomic pipelines can lead to radically different results.
Fungi identification	FindFungi,^50^ HumanMycobiomeScan,^51^ CCMetagen.^52^	Early work indicates that Fungi may play a role in IBD’s onset and progression.^53–56^	These methods are largely untested on IBD datasets. Most data on the IBD mycobiome has been analyzed using custom pipelines.
Viral identification	PhiSpy,^57^ VirSorter,^58^ PHASTER.^59^	Recent work shows that the IBD virome may show increased viral diversity and abundance.^60–67^	These methods are largely untested on IBD datasets. Most data on the IBD virome has been analyzed using custom pipelines.
Microbial network analysis	SparCC,^68^ SPIEC-EASI^69^ and MENAP,^70^ MDSINE.^71^	Network analysis implicated *E.coli* and *O. formigenes* as IBD-associated taxa.^72^	The use of cross-sectional data can mean that microbial networks in different states may be compared and combined into a single network. Time series data allows the application of the Lotka-Volterra framework, though this approach is vulnerable to changes in absolute abundance.

## Novel approaches to characterize the microbiome and its products

### Combining flow cytometry with high-throughput sequencing

While the analysis of microbial components of the human microbiome can offer important insights into the etiology of IBD, changes in the abundance of specific bacteria do not necessarily reflect the immunological impact of the microbiome on the host. Fluorescence-activated cell sorting (FACS) coupled with 16S rRNA gene sequencing is a powerful flow cytometry-based technology that allows quantification and sorting of bacteria coated with immunoglobulin A antibodies (IgA-Seq).^6–10^ Secretory IgA (sIgA) is the predominant antibody isotype at mucosal surfaces, and plays a direct role in intestinal immunity. Large amounts of sIgA are produced by plasma cells located in Peyer patches, and secreted into the intestinal lumen by transcytosis, where it coats invading pathogens with high affinity, blocking their binding to receptors on epithelial cells and facilitating their removal by immune exclusion.^73,74^ Simultaneously, sIgA maintains homeostasis by coating commensals in what is generally considered a relatively low-affinity and specificity interaction, containing commensals within the gut, regulating their composition, gene expression, and motility.^73,75–78^

By using labeled anti-human IgA antibodies, gut bacteria can be sorted into IgA-coated (IgA+) and non-coated (IgA-) fractions. Compositional profiles of the sorted bacterial fractions are then identified with 16S rRNA gene sequencing. IgA-Seq-based studies have identified aberrant sIgA-microbiota interactions in IBD. In particular, increased levels of sIgA coating and differential binding of pathobionts have been shown, compared to healthy controls,^7,11,79,80^ supporting the potential of this technology to identify immunologically reactive microbiota associated with disease. Some studies have further shown that many of the IgA responses to the microbiome seem to be strain-specific. Yang and colleagues showed that different strains of *Bacteroides ovatus* can drive either high or low mucosal IgA production in mice mono-colonized with different human fecal strains.^81^ Similarly, Palm and collaborators isolated two strains of *B. fragilis* from IBD patients that showed either high or low IgA coating, and used them to colonize germ-free mice with induced colitis. Results showed that the IgA+ strain exacerbated inflammation and reduced colon length, compared to the IgA- strain.^7^ Viladomiu and colleagues identified adherent- invasive *E. coli* (AIEC) strains in Crohn’s disease (CD) with and without associated spondyloarthritis (SpA).^79^ Although no significant differences were observed in terms of relative abundance between both groups, IgA+ *E. coli* was significantly enriched in CD patients with SpA, and those strains were also enriched in virulence genes, compared to CD strains from non-SpA patients. These findings reinforce the idea that IgA-coating may not be dependent on bacterial abundances in the gut, but rather is directed to distinct immunologically reactive strains.^7,9,79–81^

One of the main advantages of this methodology is its capacity to recover organisms that elicit IgA responses, which allow for further experimental validation such as testing the immunological impact of identified bacteria in germ-free mice or *in vitro* cell models.^7,9,79,81,82^ These experiments have shown that specific, highly immune-reactive members of the human microbiota confer susceptibility to IBD upon transplantation into IBD-models of germ-free mice. In the study by Palm and colleagues, authors sorted IgA± from IBD patients, then isolated and rationally selected bacterial taxa based on their level of IgA coating. They then assembled highly coated and low-coated bacterial consortia and used them to colonize germ-free mice. Results showed a dramatic increase in intestinal inflammation and susceptibility to induced colitis when mice were exposed to the highly IgA+ consortium.^7^ On the other hand, Viladomiu and colleagues used an *in vitro* model of Caco-2 intestinal epithelial cells to perform functional analysis of IgA-coated *E. coli* isolated from CD-SpA. This revealed the strain’s high adherence and invasion capability, as well as persistence following macrophage invasion, consistent with its characterization as adherent-invasive *E.coli*.^79^ Authors also exposed germ-free mice to the highly invasive *E. coli*, which was able to induce Th17 mucosal immunity. They further showed that the same *E. coli* strain aggravated colitis or inflammatory arthritis after transplantation into interleukin-10 deficient or K/BxN mice, respectively. Finally, a recent study has also shown promising applications of IgA-Seq at identifying changes in IgA-binding profiles in response to IBD treatments. They found that IgA-coated taxa were predictive of progression to surgical resection.^80^

This promising technology, however, suffers from some limitations. Flow-cytometry is based on the optical properties of the sample and relies on the use of fluorescent antibodies and other cell markers that can result in nonspecific binding and differential-staining biases, which could affect IgA-Seq results and interpretation. To provide robust evidence for the specificity of the antibody to its target, antibody validation is recommended. Several methods are available for this purpose, including the use of positive and negative cells (i.e. testing the antibody against microbial communities from human samples vs microbial cultures where human IgA antibodies are not present, such as pure microbial cultures). In addition, when performing multicolor fluorescence assays (e.g. combining antibodies and viability dyes), fluorescent compensation should be applied. The principle of compensation is simple: for each fluorophore used in the experiment, a single-stained cell or bead sample must also be prepared. This process identifies spectral overlap between fluorophores and allows correction for signal “spillover” into other channels. In addition, a suspension of single cells is required for this technique to work accurately, as the detection of fluorescent signal from a cell forming part of an aggregate could result in the sorting of other non-positive particles, which could lead to misleading results. However, microbes often grow as aggregates or part of a biofilm, so filtering of larger particles and disaggregation through shaking or sonication are needed before sorting. Moreover, a high risk of microbial contamination within the cytometer is likely to occur if thorough cleaning and de-contamination protocols are not established. Importantly, software pipelines for microbiome analysis do not currently incorporate functionality specific for IgA-Seq data analysis, which can preclude the standardization of results. On the other hand, identification of disease-associated members of the microbiota can be hindered by the cross-reactivity of antibodies toward both pathobionts and commensals. The specificity of antibodies to microbial antigens and the underlying mechanisms by which the immune system distinguishes pathogens from commensals remains largely unknown.^8,82,83^ Thus, comparison of IgA-coating patterns between disease and healthy controls seems imperative, which requires well-described IgA-coating profiles in healthy conditions before this methodology can be applied as a diagnostic tool. Besides IgA, other immunoglobulin isotypes are found in the gut lumen. In particular, previous studies have shown that IgG and IgM antibodies also bind differentially to the microbiota,^82,84,85^ and increased levels in IgG-coating have been observed in CD and UC.^79,82,86^ In a recent study, UC was associated with increased levels in both IgA- and IgG-bound bacteria, whereas only an increase in IgG was observed in CD, suggesting differential patterns of IgA- and IgG-binding to fecal bacteria in UC and CD.^11^

In addition to bacteria, it has recently been shown that intestinal fungi can also be targeted by secretory IgA and systemic IgG and IgM.^14,87,88^ Doron and colleagues sorted and sequenced IgA and IgG antibody-bound fungal cells from the stool of healthy individuals, which identified *Candida albicans* as the most immunoglobulin-reactive species in healthy conditions.^88^ Subsequent studies identified that IgA preferentially targets fungal hyphal morphologies that are associated with invasion and virulence, rather than yeast morphologies, suggesting that IgA promotes a mutualistic relationship between the host and commensal fungi.^14,87^ Interestingly, Doron and collaborators showed a dysregulated antibody response against hyphal morphotypes of *C. albicans*, and an overall increase in fungal hyphal morphotypes in intestinal mucosal washings from CD patients. These results suggest that IgA mechanisms targeting fungal virulence factors could be disrupted in CD, and that Ig-SEQ also has potential applications for the analysis of mycobiota involvement in the pathogenesis of IBD.^14^

### Leveraging metabolomics to study microbial metabolites and molecules

Members of the microbiome interact with the host through metabolites, both self-synthesized *de novo*, or produced by modulating human- and diet-derived components.^25^ Studying the metabolome, the combination of host and microbial-derived metabolic components, can offer important insights into molecular mechanisms observed in the gut microbiome of IBD patients. The most common approaches used to characterize the metabolome are separation of molecules by chromatography (mainly gas, GC, or liquid, LC), capillary electrophoresis (CE) with subsequent detection by mass spectrometry (MS), or nuclear magnetic resonance (NMR). All can be performed using untargeted or targeted methods, offering unique advantages and disadvantages.^26^ In untargeted approaches, a complete profile of all the detectable metabolites present in the sample of interest can be obtained. Targeted methods, on the other hand, aim at identifying specific molecules from a predefined panel of targets. While targeted mass spectrometry offers better specificity and provides absolute quantification, this is at the expense of lower sensitivity (i.e. only metabolites in the panel can be measured), and requires *a priori* knowledge about what metabolites to target.^26^ Untargeted metabolomics, on the other hand, can quantify a vast number of uncharacterized metabolites in a single run, which allows the identification of known and unknown molecules in the sample of interest, and is particularly useful for biomarker discovery.^15,26^ However, as only a subset of the metabolome is annotated, this method is generally applied to compare profiles of groups of samples. To resolve some of these issues, additional experimental and analytical approaches have been proposed, such as validation of metabolites against compound libraries, the use of *ad hoc* analytical pipelines to enhance metabolite clustering and annotation,^15^ or the application of methods that couple targeted an untargeted metabolomics.^26^ Although metabolomics theoretically identifies all small molecules present in a sample, in practice this is limited by how molecules are extracted, ionized, and detected. In addition, the different types of metabolomic tools provide different levels of resolution and thus the choice of analytical methods can produce different results. Therefore, the combination of complementary instrumental platforms and analytical methods is required for a comprehensive profiling of the metabolome.^25^

IBD is associated with an aberrant microbiome that has a reduced capacity to produce metabolites that are important for intestinal homeostasis, while producing pathogenic molecules that trigger pro-inflammatory processes in the host. Integrating metabolomic and metagenomic profiling has allowed the identification of metabolite biomarkers that differentiate IBD patients and healthy controls.^15,89^ Such differences and the knowledge that specific microbial-derived metabolites promote intestinal barrier integrity and regulate inflammatory processes have inspired numerous translational studies seeking to apply microbiome-derived metabolites in novel therapeutics for IBD.^90^

### Short-chain fatty acids

Short-chain fatty acids (SCFA), including butyrate, acetate, and propionate, are produced by gut bacteria through the digestion of dietary fibers. These metabolites participate in numerous cellular and immunological processes, such as stimulating the production of mucins, reducing intestinal permeability, and promoting anti-inflammatory pathways.^27,33^ Both gas chromatography – in combination with various detectors – and liquid chromatography are commonly used to analyze SCFA in fecal samples.^28,29^ However, these methods have low sensitivity for the detection of SCFA in complex samples, due to the small mass of the compounds and spectral overlap with interfering peaks from solvents and additives. In addition, SCFA’s hydrophilicity results in poor chromatographic separation. To overcome these problems, SCFA can be derivatized, a process that alters the structure of the analyte to facilitate its isolation, separation, or detection.^27–29^ Nevertheless, most chemical derivatization procedures for GC/MS and LC/MS are not suitable for the analysis of SCFA in biological samples due to their volatility and thermal instability. Consequently, several protocols have been specifically adapted for metabolomic analysis of SCFA, including chemical derivatization methods,^28,30^ as well as isotopically labeled derivatization methods, which introduce isotope tags for accurate compound quantification while improving the chromatographic retention of metabolites.^27,31,32^

It has been observed that levels of butyrate and their bacterial producers are lower in patients with active IBD compared to controls^16–18,89,91^. This suggests that restoring gut microbial homeostasis can increase luminal levels of butyrate and exert an anti-inflammatory function. However, the complex ecology of the gut microbiome makes it difficult to modulate the butyrate-producing capacity of these communities, and direct supplementation of SCFAs has been explored as a more practical alternative. Butyrate administration has shown some promise in the treatment of IBD, including reduction of inflammation, symptoms and development of the disease.^34–36^ *In vivo* and *in vitro* studies have shown that butyrate’s anti-inflammatory properties are associated with the inhibition of nuclear factor kappa B, which reduces the expression of pro-inflammatory genes.^36,92,93^ In addition, several studies have shown that the combination of butyrate with biologics can significantly improve their therapeutic effect. In one study, mesalazine (5-aminosalicylic acid, 5-ASA) and butyrate significantly ameliorated disease based on endoscopic and histologic scores, and maintained clinical remission compared with treatment with isolated 5-ASA alone.^34,35,94^ The levels of butyrate and substrates involved in butyrate synthesis have also been associated with anti-TNFα therapy efficacy in IBD patients.^91^ Multiple clinical studies are currently investigating the efficacy of SCFA in the treatment of IBD, including the administration of encapsulated butyrate to reduce intestinal inflammation in IBD and irritable bowel syndrome patients (www.clinicaltrials.gov: NCT04879914), butyrate in combination with other therapeutics (e.g. hydroxocobalamin to treat UC, trial NCT04259060), or dietary interventions to modulate the microbiome and increase the amount of colonic SCFA (NCT04520594, NCT04522271, and NCT04065048).

### Dietary amino acids

IBD is also characterized by a reduction in specific dietary amino acids that are affected by microbial activity.^20,21^ An important example is tryptophan (Trp), which serves as substrate to gut microbial enzymes that convert dietary Trp into tryptamine and other indole derivatives. Trp derivatives can function as endogenous ligands for the aryl hydrocarbon receptor (AhR), that is essential to maintain gut homeostasis and immune responses.^21,22^ Therefore, a reduction of Trp and other Trp-derived AhR ligands can have important effects on the anti-inflammatory and protective functions associated to AhR activity. In a murine model of dextran sulfate sodium (DSS)-induced colitis, administration of a Trp-deficient diet or depletion of AhR encoding genes exacerbated colitis and led to weight loss and reduction of the production of antimicrobial peptides and other important chemokines.^95,96^ Administration of Trp in the diet attenuates inflammation and protects against colitis symptoms. Similar results have been reported in other studies with DSS-induced colitis animal models treated with Trp or its derivatives.^96–99^ Among the Trp-derivative metabolites that can ameliorate induced colitis in animals are the microbially produced indole (IND) and indole-3-propionate (IPA), which have been shown to regulate intestinal epithelial function via activation of IL-10 expression.^97,100^ Other derivatives, such as indole-3-pyruvate (IPyA), indole-3-aldehyde (I3A), and indole-3-ethanol (I3E) are essential to modulate gut barrier integrity via tight junctions and adherent junctions.^21,99^ Scott and colleagues demonstrated that IPyA, I3A, and I3E protected against intestinal permeability and colitis in mice, and that removal of Trp-metabolizing bacteria by antibiotic treatment depleted their production.^99^ Therefore, bacterial tryptophan metabolites have an important potential use as treatments for IBD symptoms, although evidence from clinical studies in humans is not yet available.

LC-MS/MS has been widely used to quantify Trp and its metabolites.^38,101^ However, this method often focuses only on major Trp metabolites, such as kynurenine and tryptophan. Nevertheless, the majority of Trp is metabolized through the kynurenine pathway into a variety of less-abundant compounds, and changes in concentrations of these less-abundant metabolites have been observed in IBD.^20,38^ Whiley and colleagues developed a specific method to quantify Trp and its downstream metabolites, called targeted ultrahigh-performance liquid chromatography tandem mass spectrometry with electrospray ionization (UHPLC-ESI-MS/MS), and validated it in a cohort of UC patients.^37^ Besides Trp, the metabolism of other amino acids, such as arginine and glutamine, are also altered in IBD and could represent additional targets to treat the disease.^21^ However, findings from studies in animal models have not been able to be replicated in human studies in pediatric CD, adult CD and UC patients.^102,103^

### Secondary bile acids

Secondary bile acids (SBA) are also bacterial-derived gut metabolites, which play key roles as signaling molecules.^104^ While primary bile acids (PBA) are produced from cholesterol in the liver, microorganisms are responsible for transforming them into secondary and tertiary bile acids in the gut via deconjugation of glycine or taurine by bile salt hydrolases, and dehydroxylation, oxidation, and epimerization of the cholesterol core.^105^ Alterations in the chemistry of SBA have been observed in IBD patients.^15,23,24^ For example, decreased bile acid deconjugation has been associated with UC and CD, and the alterations in bile acid metabolism have been directly linked with the microbial dysbiosis occurring in IBD.^106^ Sinha and collaborators recently combined metabolomic, microbiome, metagenomic, and transcriptomic profiling of stool from ileal pouches in colectomy-treated UC patients. Results showed that, compared to controls, patients had reduced levels of the two most abundant SBA in the gut, lithocholic acid (LCA) and deoxycholic acid (DCA), as well as genes involved in SBA transformation from PBA, and reduced abundance of Ruminococcaceae, a family of bacteria known to produce SBA.^24^ Although approximately 26% of bacterial strains identified in the human gut contain bile salt hydrolases that can deconjugate PBA, only a few (from the Lachnospiraceae and Ruminococcaceae families) are currently known to perform subsequent dehydroxylation to generate DCA and LCA.^105,107^

Therapeutics targeting receptors involved in bile acid metabolism can affect microbiome composition.^108,109^ In parallel, the expression of ileal bile acid transporters, particularly the main transporter, apical sodium-dependent bile acid transporter (ASBT), have been shown to be decreased in animal models of intestinal inflammation, as well as in IBD patients.^23,110–112^ Therefore, the restoration of bile acid metabolism is gaining attention for its therapeutic potential. Clinical studies have demonstrated that patients with bile acid malabsorption (BAM) associated diarrhea respond well to some bile acid sequestering agents, including cholestyramine, colestipol, or colesevelam.^110,113,114^ Colesevelam therapy has also been shown to be effective at reducing the number of liquid stools/day, improving stool consistency in CD patients, and treating post-operative BAM in CD compared to other therapeutics.^114,115^ Because bile acid sequestrants are not absorbed in the intestine, systemic effects are generally low. However, they can bind to other vitamins and drugs and interfere with their absorption, which could result in vitamin deficiencies and inefficacy of other treatments.^115^

Promising results have also been observed with direct administration of SBA in experimental models of colitis. However, an important limitation of the oral administration of SBA is their rapid uptake into the enterohepatic circulation in the small intestine and delivery to the liver, which can result in low concentrations reaching the colon.^108,116^ Therefore, alternative delivery methods may be required to achieve therapeutic concentration in the colon. Recently, rectal administration of DCA and LCA mitigated inflammation in three different murine models of acute and chronic colitis, and reduced the expression of key pro-inflammatory cytokines and chemokines.^24^ DCA and LCA were shown to exert their effects via activation of the widely expressed G protein-coupled receptor TGR5. Together with TGR5, the farnesoid X receptor (FXR) were both activated by bile salts and involved in immune modulation and intestinal barrier functions. Several FXR-agonists are being explored as novel therapeutics to treat intestinal inflammatory conditions. Among them, ursodeoxycholic acid (UDCA) is an SBA with cytoprotective and immunomodulatory properties that is currently used to treat certain types of cholestasis and primary biliary cirrhosis.^116^ UDCA therapies have also shown anti-inflammatory effects in animal models of colitis,^117^ which supports the idea that UDCA may also be useful in preventing or treating IBD in humans. This hypothesis is currently being tested in a phase-II clinical trial that investigates the ability of UDCA to reduce inflammation and improve quality of life in UC pouch patients (NCT03724175). UDCA can also modify the microbiome: a clinical trial of long-term (3 years) treatment with UDCA to prevent colorectal adenomatous polyps resulted in an overrepresentation of *F. prausnitzii* and underrepresentation of *R. gnavus*, two species that show abnormally low and high levels in IBD, respectively.^109^ Given the clear association between microbial imbalances and bile acid metabolism, approaches that seek to restore key bacterial species (e.g. SBA-producers) and bile acid metabolism hold great clinical promise in the treatment of IBD.

### Bacterial cell components

Bacterial cell components may also be used as additional targets to treat intestinal inflammatory conditions. Capsular polysaccharide A (PSA), found on the cell surface of the gut commensal *Bacteroides fragilis*, has been extensively studied for its immune modulatory properties and its key role in reducing inflammation.^118^ Colonization of mice with PSA-producer strains of *B. fragilis* or with purified PSA can protect animals against experimentally induced colitis and cure the disease. The mechanism of action of PSA involves the induction of IL-10-secreting T_reg_s mediated by TLR2 signaling.^119,120^ In a recent study, the zwitterionic polysaccharide TP2, a capsular polysaccharide of *B. fragilis,* was extracted and administered to rats with induced enteritis.^121^ Treatment with TP2 significantly alleviated inflammation and reduced adhesion score and ulcer incidence. In addition, TP2 was not degraded during passage through the intestinal tract in models *in vivo* and *in vitro* simulating gastric, intestinal, and colonic conditions. TP2 integrity was maintained during absorption, distribution, metabolism, and excretion. However, safety and pharmacokinetic studies of PSA and other capsular bacterial polysaccharides for its use in humans are not yet available.

Although the characterization of microbial-derived metabolites and molecules in IBD is a promising research approach, their application encompasses some drawbacks. Metabolite biomarkers of IBD are commonly identified from fecal samples, but as they undergo different processes of absorption and metabolic changes before being excreted, they may not be the same molecules involved in relevant host interactions. Further, the use of single therapeutic metabolites has important pharmacological limitations related to dose, administration, and metabolic stability.^90^ The use of crude metabolites can often lead to high variability in the amounts that are able to reach the target site. If administered systematically, there is a risk that the metabolite does not reach its target, as the serum half-life of certain molecules can be very short. Oral administration is an attractive alternative, although as soluble metabolites pass through the gastro-intestinal tract they can be partially broken down by digestive enzymes, which makes it difficult to estimate how much product reaches the gut and complicates reproducibility. Additional studies are thus required that accurately estimate the efficacy of microbial-derived molecules and their optimal method of administration for the treatment of IBD.

## Analyzing microbiome data in IBD

### Identifying IBD-associated bacterial strains

It has been hypothesized that, similar to Koch’s postulates, the identification of strain-level variation in IBD patients has the potential to reveal novel insights into its pathogenicity and pave the way to new treatments.^122,123^ This has proven true in the case of *E. coli*,^124–128^ where certain strains have indeed been associated with IBD. The advent of high-throughput sequencing has provided a novel approach to identify IBD-associated strains through the analysis of shotgun metagenomic data.

More recent work has implicated additional strains in IBD pathogenesis. In a study of IBD twins,^46^ eight species were found to have higher relative abundance in IBD-twins relative to healthy controls, as well as between healthy co-twins and healthy controls, including *Gordonibacter pamelaeae, Escherichia unclassified*, and *Eggerthella unclassified*.^46^ Multiple studies have shown that *Ruminococcus gnavus* appears more frequently in IBD patients,^15,47^ particularly in CD patients. In a study of IBD patients receiving anti-integrin treatment, *Roseburia inulinivorans* and *Burkholderiales* species were found in significantly higher abundance in CD patients achieving week 14 remission compared to patients who did not enter remission,^49^ suggesting that bacterial species and strains could also play an important role in disease progression.

An important caveat of these studies is that multiple software pipelines exist for identifying bacterial taxa at high resolution from metagenomic data^129^ and the interpretation of these results depends significantly on the specific tool used.^42,130,131^ While some tools construct k-mers and assemble them using de Bruijn graphs,^132^ others assign reads to specific genomes using a set of marker genes.^39–41^ While computationally less expensive, this marker-based approach depends strongly on the reference database used. Methods such as MetaPhlAn have been often utilized in IBD studies,^15,46–49^ and although recently expanded with the inclusion of additional genomes,^39^ it remains to be determined to what extent these results are limited by existing genome collections. Recently developed methods based on strain collections^42^ have increased potential to identify strains with high confidence and resolution, although they also suffer from limitations related to the size of the collection being used.

Overall, the use and development of software tools to identify bacterial strains from shotgun metagenomic data shows great promise to characterize potentially pathogenic microbes in IBD in a high-throughput manner and without the limitations of approaches that require microbial isolation. However, the variety of analysis pipelines and the lack of a gold standard to compare them suggests the variability of strains associated with IBD could not only be due to cohort-specific factors, but also partially driven by the analysis itself. Thus, methods that compare and validate multiple-software tools are needed.

### The temporal variability of the IBD microbiome

IBD is a chronic disease that undergoes different stages during its natural history, with periods of quiescence punctuated by acute inflammatory episodes (“flares”). Patients often require partial or total colectomy, which can happen several years after the disease is initially diagnosed. The microbiome of IBD patients differs with that of healthy subjects,^133,134^ varies with IBD type,^18^ and treatment.^12,135–139^ Moreover, the history of the patient’s treatment course matters in their response to future treatments.^135^ This rising appreciation of the temporal variability of the disease has led to a call for longitudinal studies.^138,140,141^

Some studies have partially characterized temporal changes in cohorts of IBD patients,^142,143^ in mouse models,^60,144–147^ and during flares.^148^ However, many of these have been exploratory studies, with either small patient cohorts^139,143,149^ or large cohorts but with limited sampling.^150^ Temporal sampling differs significantly between studies ranging from weekly,^151^ or monthly,^18^ to annual or longer intervals between samples,^133,136,150^ which further complicates analysis and interpretation. Some results suggest that daily sampling is not necessary to fully characterize the temporal variability of microbial communities, and that samples taken every few weeks may be enough to capture disease-relevant information.^152^ It is, however, unclear how frequent sampling the needs to be to capture features of clinical importance, such as the prediction of flares, the need to escalate therapy, or the future likelihood of requiring surgery.

Importantly, most longitudinal studies of microbiome variation in IBD bin temporal changes into categories so that comparisons can be made at the group level. The intent here is to determine which taxa are higher in healthy subjects compared to IBD patients. This approach has facilitated the characterization of temporal variation in IBD.^19,61,62,153,154^ It has identified signals associated with response to anti-TNF therapy,^19^ as well as the likelihood of successful clinical and histological remission of UC after FMT.^62^ However, this approach also limits our ability to uncover subject-specific characteristics of the disease or the likelihood a specific patient will respond to treatment, one of the premises of personalized medicine. Therefore, new methodologies that allow the analysis of longitudinal microbiome data at the subject level are an important unmet need that will be required for better patient stratification and treatment efficacy.

### Fungi and viruses: overlooked contributors in IBD studies

Most studies exploring the gut microbiome have focused their attention in the bacterial component, largely overlooking the fungal (mycobiome) and viral (virome) elements. Viruses and fungi also play important roles in the intestinal ecosystem, and they are now receiving increased attention in the context of human health and disease.^63–67^

Albeit essential to human health, the mycobiome comprises a lower proportion of the overall microbiome compared to bacteria and viruses,^66^ which partially explains why most IBD mycobiome sequencing studies to date are based on amplicon sequencing.^53–56^ Two ribosomal regions have been commonly used in mycobiome analyses: the 18S rRNA gene and the internal transcribed spacer (ITS) regions, internal to the 18S, 5.8S, and 28S rRNA sequences.^66,155^ The ITS regions have been identified as the most effective to characterize fungi in complex communities, and are currently used as the primary approach for fungal analyses.^66,156^ ITS regions are present in high copy number and are conserved across fungi, but contain two highly variable fragments, ITS1 and ITS2, that, combined with the low variable 5.8S fragment, improves resolution at low taxonomic levels. The use of ITS regions, however, does not come without complications, including amplification^67^ and sequencing biases.^157,158^ While shotgun sequencing offers multiple advantages over amplicon-based approaches (improved taxonomic resolution, no amplification biases, functional information), the number of fungal metagenomic studies in IBD are few,^66,137^ partially due to the inherent difficulty of working with the low amounts of fungal (compared to bacterial or viral) DNA present in stool.

The number of research studies interrogating the IBD virome are also scarce, but there is growing evidence that suggests that IBD viral communities also exhibit differences in composition, with increased viral diversity and abundance.^64,159–165^ The viral component of the microbiota is mainly composed of prokaryotic-infecting viruses (bacteriophages or phages),^64,65,166^ which have the ability to shape bacterial communities and can have important repercussions in IBD.^65,159,167,168^

The vast diversity of viruses and their variability in morphology, genetic material (DNA or RNA), configuration (single- or double-stranded), and genes sense of orientation represent important limitations for the study of viral communities.^169,170^ Phages can also vary depending on their interactions with the bacterial host, being able to infect as lytic or temperate phages.^170^ As viruses do not have a single, universal marker gene, studying the virome relies heavily in non-targeted, shotgun sequencing approaches.^170,171^ Although the amounts of viral particles in the gut is estimated to be at least equal to the number of bacterial cells, the size of viral genomes is smaller and viral nucleic acids only account for a small proportion of the gut microbiome.^166,170,172^ Thus, untargeted shotgun metagenomic sequencing methods often fail to yield sufficient viral reads. To overcome this problem, viral particles are usually concentrated and purified before performing sequencing.^160,164,173^ In some cases, however, absolute yields of viral nucleic acid preparations are low and efforts to specifically favor viral yields still fail to deliver sufficient information. For example, Lloyd-Price and colleagues performed multi-omic analyses on various samples from IBD patients, obtaining an in-depth profile of the bacterial communities, and at the same time, purified viral RNA from stool, which was amplified and subsequently sequenced. Despite the efforts to investigate the virome, only a small number of viruses could be identified, and only a single phage could be identified as being differentially enriched in IBD and non-IBD dysbiosis.^89^ Alternatively, methods such as Multiple Displacement Amplification (MDA) can increase viral and fungal genomic concentrations by performing whole-genome amplification without sequence-specific primers prior to shotgun sequencing.^65,174,175^ However, MDA is a nonspecific reaction that can amplify any DNA present in the sample, including contaminant DNA from reagents or the environment.

An additional challenge in fungal and viral analysis is that residual genomic material from human and bacterial cells are also extracted and need to be filtered out prior to analysis. Removal of host-contaminant sequences can be achieved by mapping the reads against reference genomes with computational tools like Bowtie,^176^ DeconSeq,^177^ BBMap (https://sourceforge.net/projects/bbmap/), and BMTagger (ftp://ftp.ncbi.nlm.nih.gov/pub/agarwala/bmtagger/). Tools such as ViromeQC can be used to quantify bacterial, fungal, and archaeal contaminations in virome sequencing data.^178^ Specific tools for viral identification in metagenomic data are also available, including PhiSpy,^57^ VirSorter,^58^ and PHASTER.^59^ Similarly, tools to identify fungal sequences in metagenomic datasets include FindFungi,^50^ HumanMycobiomeScan,^51^ and CCMetagen,^52^ which can identify reads originating from eukaryotic and prokaryotic genomes.

Finally, comprehensive pipelines for decoding, quality filtering, sequence clustering, and taxonomic assignment of high-throughput sequencing data are also skewed towards bacterial analyses. QIIME, RDP, or Mothur can be adapted to incorporate fungal analysis. Other software pipelines exclusive for fungal analysis include BROCC (http://sourceforge.net/projects/brocc/), CloVR-ITS^179^ or PIPITS.^180^ On top of *ad hoc* software tools and algorithms for viral contig annotation and viral databases for sequencing data processing, recent stand-alone pipelines specific to viral metagenomic analysis have been developed, such as ViroMatch,^181^ Sunbeam,^182^ and VirusSeeker.^183^

### Microbial networks and compositional data analysis

The microbiome is a complex ecosystem, composed of many interacting entities that form a dynamic community.^184^ Networks built to represent such high-dimensional systems are powerful tools because they can capture putative relationships between members of the community that might be predictive of outcomes not associated with individual components of the network. The construction and analysis of microbiome networks, however, requires approaches specifically designed for them, given the sparse and compositional nature of the data.^185^ Microbiome data is sparse since most samples only contain a small subset of all possible taxa. It is also compositional, as data is often expressed as relative – as opposed to absolute – abundances.^186^ Because compositional data must add up to unity, an increase in the relative abundance of one taxon induces a decrease in relative abundance of other taxa, even when the absolute number of those taxa have not changed.

Compositionality is partially a consequence of the rarefaction process performed to normalize data across samples and groups.^187^ While there is no consensus on the extent to which rarefaction techniques might reduce statistical power,^188,189^ compositional data introduces important biases in the analysis of correlations or when identifying differentially abundant features, and can result in incorrect conclusions if not properly accounted for.^190,191^ Several recent methods have been developed to analyze microbiome data in a compositional context. The use of a proportionality statistic based on log-ratios has been shown to estimate when variables are proportional in an accurate manner,^190^ and reference frames have also been proposed for differential abundance analysis.^192^ The sparse nature of microbiome data can also lead to a large number of false correlations driven by influential observations. We have recently proposed an approach based on jackknifing that significantly reduces the number of such false positives.^193^

Among the most commonly used methods for network analysis in microbiome data are SparCC,^68^ SPIEC-EASI^69^ and MENAP.^70^ SparCC uses log-transformed data to infer co-occurrence relationships between taxa. SPIEC-EASI employs a sparse neighborhood and inverse covariance selection framework to determine the underlying ecological network. MENAP uses a Random Matrix Theory-based approach to determine the adjacency matrix used to construct the network. Other tools such as ReBoot/CoNet,^194^ REBACCA,^195^ CCLasso,^196^ Mint,^197^ gCoda,^198^ have emerged more recently, and additional work has been proposed to construct microbe–metabolite interaction networks.^199^ When longitudinal data is available, ecological network characterization using the Lotka-Volterra framework has provided novel insights into *Clostridium difficile* colonization,^200^ microbial and immune cell interactions,^201^ and inter-kingdom interactions in the infant microbiome.^202^ This approach has been implemented in MDSINE, a suite of algorithms for microbiome time-series data analysis and prediction.^71^ Overall, the application of network analysis methods to IBD data is still in early stages. Recent work combining SparCC and SPIEC-EASI on IBD patients and healthy controls showed that a signature of IBD can be reflected in microbial co-abundance analysis, particularly implicating *E. coli* and *O. formigenes* as IBD-associated taxa.^72^

While the use of these methods can partially reduce some of the complexities associated with compositionality, they are not without issues. Log-ratio approaches cannot work with zeroes, which must be replaced by pseudo-counts, introducing additional biases.^203^ Further, the lack of information on microbial load when using relative abundances is critical in most microbiology studies^204^ and limits the application of tools like ecological network modeling. The use of methods to quantify absolute counts is an alternative to relative abundance-based methods that should be considered, as they have been shown to be of high relevance in microbiome studies of IBD.^205^

## Therapeutic engineering of the microbiome

### Microbial transplants: a promising therapeutic approach

**Figure 1.**
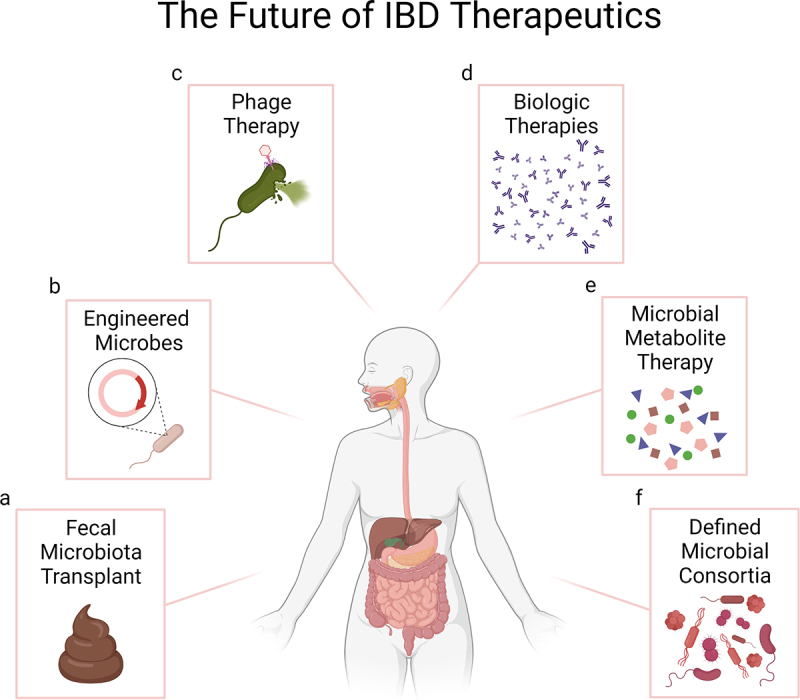
Opportunities for microbiome therapeutics in IBD. **a**. Fecal Microbiota Transplant (FMT) transplants whole microbial communities from healthy donors into IBD patients and has shown promising results particularly in the treatment of UC. **b**. Bacterial engineering can be used to enhance the beneficial properties of bacterial strains, including targeted delivery of therapeutic molecules, which offers important efficacy and safety advantages compared to the use of FMTs or molecules delivered systemically. **c**. Phage therapy or “decolonization”, currently being explored for CD and UC, selectively targets and removes specific bacteria associated with disease, without disrupting other members of the gut microbiota. **d**. Biologic therapies, which can induce clinical and histological healing, are the current standard of care in IBD. Lack of response, development of resistance and side effects remain challenges for some patients. **e**. The aberrant microbiome of IBD patients has a reduced capacity to produce metabolites that modulate intestinal homeostasis. Direct administration or modulation of the microbiome to enhance the production of such metabolites is being explored as potential therapeutics for IBD. **f**. Microbial consortia designed to induce specific immune responses are also being investigated as therapeutics in animal models and pioneered in human studies. Image created with BioRender.com.

Among the different approaches to treat IBD, microbiome-based therapeutics have gained tremendous attention as a novel approach to efficiently induce remission (Figure 1). Although pre- and probiotics have been utilized in the past with mixed results,^4^ fecal microbiota transplants (FMTs) are arguably a more commonly tested method in current research and clinical studies. The extremely high efficacy of FMTs in recurrent *Clostridioides difficile* infection (CDI) refractory to antibiotics^206–209^ has paved the way for their use in the treatment of IBD. Moayyedi and colleagues demonstrated how FMTs can induce remission at 7 weeks post-FMT in ulcerative colitis (UC).^210^ Intriguingly, 7 of the 9 patients who responded to treatment had received fecal material from a single donor, suggesting that the microbiome of this donor was different from that of other donors and particularly efficient at inducing remission. A later study of an intense FMT regime demonstrated remission in 27% of UC patients, compared to only 8% with placebo.^211^ No donor-specific effects were noted, and several bacterial and metabolite biomarkers could be identified to be associated with remission.^62^

While results of FMTs in IBD are highly encouraging, there are issues that moderate the general applicability of this approach. Safety is commonly discussed as an important limitation of FMTs, given the risk of transferring pathogenic agents to the recipient.^212^ The global COVID-19 pandemic has further stressed the need to rigorously test fecal material prior to transplantation. The use of a procedure that is still experimental requires a careful assessment of risks and benefits for different patient cohorts. A more rigorous exclusion criteria could potentially prevent or limit the severity of adverse events in future studies. Indeed, a nation-wide study of FMT in CDI has recently shown the procedure to have a good safety profile.^213^ However, the invasive nature of the procedure and its associated economic cost are disadvantages compared to other therapies. Finally, the heterogeneity of the product used in FMTs presents two important challenges: because the fecal material varies donor to donor, response to intervention (or lack of it) depends both on patient and donor, which complicates interpretation of results. It is also unclear how fecal material can be regulated, if at all, in terms of intellectual property and commercialization, which partially hinders the involvement of the private sector in developing FMT therapeutics.

To address some of these problems, fecal material in capsule form has been pioneered in the study of CDI.^214^ As they are taken orally, fecal pills are less invasive and more cost-efficient, at the expense of a diminished (although still high) efficacy, at least in CDI.^215^ Their efficacy in IBD, however, remains to be tested. An alternative that is gaining traction in microbial therapeutics is the use of bacterial communities, which contain defined sets of isolates with desirable properties to treat a given condition. This approach circumvents several of the main issues of solutions based on fecal material, and they provide a higher level of safety and homogeneity, although at the potential expense of having lower efficacy. A recent study of an oral formulation of defined Firmicutes spores found that, in conjunction with vancomycin, clinical response was higher than in no vancomycin plus spores.^216^ It should be noted that this study did not consider histological remission, a stricter endpoint that has been reached with full FMTs in UC,^210,217^ and that clinical remission was most notable in those patients who also received vancomycin, suggesting that efficacy of the defined community alone might be lower than that observed with FMTs.

The curation of defined microbial consortia built to prevent or treat disease has several advantages over FMTs, including replicability, safety, and scalability. Being able to select subsets of bacteria that exhibit desirable properties would be a fundamental step forward in the development of efficient microbial therapeutics.^218^ As with FMTs, the rational design of microbial consortia also has its roots in CDI studies.^219^ Recent work has demonstrated that consortia with less than 20 strains can prevent or treat colitis in murine models after two weeks of treatment.^220^ The mice further showed reduced tissue inflammation (histology, inflammatory cytokines) and increased levels of metabolites associated with a healthy mucosa, demonstrating the feasibility of this approach in a model organism. While these findings need to be replicated and likely will require refinements to be effective in humans, results suggest that remission, both clinical and histological, may be possible with defined microbial communities.

### Next-generation probiotics: genetic engineering of bacterial strains

Due to the difficulties and limited success of using single metabolites and pre- and probiotics as therapeutics for IBD, synthetic biology approaches have begun to be applied to produce engineered strains that can deliver selected therapeutic molecules, thus enhancing their probiotic capacity.^221,222^ One of the advantages of using orally administered engineered bacteria to deliver therapeutics is their ability to survive the passage through the intestinal tract while expressing the molecules of interest. This can circumvent the need for large amounts of crude metabolites that are mostly broken down by digestive enzymes. In addition, oral administration of bacteria ensures that therapeutic molecules are synthesized and delivered at the mucosal surfaces, which increases their efficacy compared to classic systemic therapies. At the same time, intestinal delivery of bacterial-derived molecules improves safety and prevents secondary effects related to systemic exposure to drugs, such as generalized immune-suppression.

Classic tools to genetically engineer bacteria use recombinant plasmids as cloning vectors to deliver genes of interest. Cloning vectors allow the production of large amounts of protein, but embody certain limitations including low efficiency of transformation and limitations of insert size. Recent discoveries and development of new tools, such as the CRISPR-Cas systems, have revolutionized the genetic engineering scene, offering an enormous potential to engineer genomes with greater efficacy than previously achieved.^223^ CRISPR (clustered regularly interspaced short palindromic repeats) and its associated Cas proteins are tools derived from the prokaryotic immune system that have been co-opted as genetic editing tools.^223,224^ This methodology has been applied to bacteria and yeasts to modify their functional repertoire, exploited for industrial applications or used for the direct removal of specific genes or pathogens.^222–225^ Among the different CRISPR systems, CRISPR-Cas9 and CRISPR-Cas12a (also known as Cpf1) are two major nucleases that have been used in bacterial genetic editing experiments. By inserting a guide RNA sequence targeting a specific region of the bacterial DNA, Cas nucleases introduce a break in the pathogen’s genome which allows the removal of specific genes or causes bacterial death.^223,226,227^ If a template DNA is provided, the genomic break introduced by the Cas nucleases can be repaired by homologous recombination, inserting the new DNA fragment into the bacterial genome.

Some of the current standardized treatments for IBD consist of systemic administration of anti-inflammatory drugs and antagonists of pro-inflammatory molecules. However, some patients do not respond to such treatments, or stop responding to treatment after some time.^228,229^ As an alternative, recombinant bacterial strains have been successfully engineered to synthesize active biotherapeutics including cytokines and immunoglobulins that can be delivered at mucosal sites. *Lactococcus lactis*, a nonpathogenic, lactic-acid bacterium commonly used in the production of fermented foods, is the gold standard for probiotic gene-editing strategies, due to its well-studied genome and its safety properties.^221^ One of the first recombinant *L. lactis*, from the pioneer work by Steidler and colleagues, was designed to treat IBD by secreting IL-10 *in-situ* in the colon in two murine models of disease.^230^ In one of the models, chronic colitis was induced by administration of DSS and the efficacy of *L. lactis*-secreting IL-10 (LL-mIL10) to treat symptoms was tested. In the second model, the ability of LL-mIL10 to prevent disease was tested in IL-10-/- mice, a model that spontaneously develops colitis. Results showed that LL-mIL10 was able to both reduce pathological symptoms and inflammation and prevent onset of colitis. In addition, the protective effect of bacterial-synthesized IL-10 performed better than parenteral administration of recombinant IL-10 and other systemic drugs as lower amounts were required to reduce inflammation. Based on these results, a small clinical trial with 10 CD patients was conducted with the purpose of testing the safety of *L. lactis* secreting human IL-10.^231^ Because of the safety concerns of using genetically engineered organisms in humans, the human IL-10 encoding cassette was used to replace a thymidylate synthase gene, essential for the growth of *L. lactis*, with the purpose of blocking its growth and preventing colonization in the host.^230,231^ An improvement in disease severity in 8 out of 10 participants encouraged a larger phase-II trial (www.clinicaltrials.gov: NCT00729872). However, no statistically significant results were observed compared to placebo treatment, which may have been related to insufficient bacterial viability or localized delivery at the site of inflammation. In another study, a *L. lactis* strain was engineered to secrete a low calcium response V (LcrV) protein, naturally present in enteropathogenic *Yersinia pseudotuberculosis* that evades the host immune system by stimulating IL-10 production and preventing inflammatory cascades. Results demonstrated the ability of LL-LrcV to treat and prevent colitis in two murine models of acute disease.^232^ Moreover, *L. lactis* strains have been engineered to secrete neutralizing anti-TNF-α nanobodies (small and highly stable single-domain antibodies) that can block TNF-α pro-inflammatory effects and ameliorate DSS-induced colitis in mice.^233^ Although *L. lactis* has been most frequently used in IBD studies, genome editing experiments aimed at engineering other intestinal bacteria are still scarce. For example, *Bacteroides* is among the most abundant genera in the human gut, and reduced relative abundances of this genera have been observed in IBD patients.^234^ However, engineering *Bacteroides* is challenging due to their high intraspecies genetic diversity and natural resistance to antibiotics that are used for genetic selection in the lab, which can result in low efficiency in genome editing experiments.^235–237^ In recent work by Zheng and colleagues, different CRISPR-Cas methods were used to edit the genome of several *Bacteroides* species.^227^ Results showed that FnCas12a (a nuclease from the Cas12a family) had the highest genome-editing efficiency across *Bacteroides* species. The efficacy of FnCas12a-engineered *Bacteroides* strains as therapeutics for IBD remains to be explored.

While most IBD therapies are aimed at modulating inflammatory pathways, approaches that restore intestinal barrier function and promote mucosal healing have been less explored. During acute flares the intestinal epithelial barrier is disrupted, exposing the gut lining to bacteria and exogenous antigens which can exacerbate inflammation and lead to systemic infections. In one study, the bacterium *L. lactis* was engineered to produce trefoil factors (TFF), which are known to promote intestinal barrier function and epithelial restitution.^238^ Orally administered TFF-producing *L. lactis* (LL-mTFF) was able to secrete TFF in the colon and prevent and heal colitis in a DSS-induced acute colitis murine model. Rectal administration of purified TFF was also able to reduce colitis symptoms, but much higher concentrations were needed and could not achieve the same results obtained with LL-mTFF. More recently, *E. coli* Nissle1917 (EcN), a well-studied probiotic strain, was engineered to secrete therapeutic matrices to promote gut epithelial integrity.^239^ Matrices, composed of nanofibers displaying TFFs, were able to protect against DSS-induced colitis in mice, by promoting mucosal healing and immunomodulation.

A limitation of using engineered bacteria as therapeutics is that they do not always necessarily reach the site of inflammation. To overcome this issue, targeting mechanisms for localized delivery of biotherapeutics at disease sites are being explored. Recently, a research group designed an invasive *L. lactis* strain containing a DNA vector, pValac:*il-10*, that was used to deliver the DNA into intestinal eukaryotic cells and treat mice with induced colitis. Once in the host cells, the eukaryotic cellular machinery expressed the ORF of interest, and translated and synthesized IL-10 in the intestine of the animals, which experienced a reduction of symptom severity and a delay in disease onset and development.^240–242^ Similarly, an EcN strain was engineered to invade the colon epithelium (and degrade later in the phagosome) and deliver a plasmid to express small hairpin RNA (shRNA) targeting tumor necrosis factor (TNF).^243^ This method reduced levels of TNF as well as inflammatory mediators. Finally, McKay and colleagues elegantly engineered commensal EcN expressing a therapeutic biologic directed toward nitric oxide (NO), a biomarker of IBD that is greatly increased in the intestine of patients compared to healthy controls,^244^ exploiting a phenomenon known as *pseudotaxis*. In this phenomenon, cell motility is dictated by the concentration of a chemical. Mimicking this natural process, authors engineered EcN to sense and accumulate at sites with high content of NO. At the same time, EcN were engineered to selectively synthesize a biologic, granulocyte macrophage‐colony stimulating factor (GM‐CSF), co-expressed with a signal molecule that would trigger the synthesis of the biologic in response to the presence of NO.^245^ This study opens new research and therapeutic possibilities by using “smart probiotic” bacteria specifically directed toward a known biomarker, which can elicit a genetic response from the engineered bacteria, triggering the synthesis of the biotherapeutic of interest at the precise site of disease or inflammation.

Despite the development of such innovative solutions and some promising results, most of the documented benefits of engineered bacteria producing therapeutic molecules are from studies based on animal models. There are some safety concerns about the use of modified bacteria in therapeutic settings, mainly due to potential exchange of genetic material with other bacteria, which may result in unexpected consequences. Therefore, the use of safe and genetically stable strains, and designs that ensure containment of strains in the intestinal environment or self-removal mechanisms should be a prioritized area of research. Ferenczi and collaborators removed more than 20% of the genome of the EcN strain used in their study, including transposons, and eliminated the recombination and conjugation abilities of the bacteria, minimizing the risk of horizontal gene transfer.^243^ Moreover, the ability of engineered bacteria to “escape” the intestinal ecosystem and invade other sites could be reduced by strategies that introduce mechanisms that block their growth or lead to bacterial self-destruction within a few days of synthesizing the therapeutic of interest.^230,231,243^ Finally, an additional limitation that remains elusive, even to engineered bacteria is the ability to control the dose effect, a problem that researchers have not yet been able to solve and which will require further research efforts.

Besides engineering individual bacteria, efforts are being made to selectively edit the intestinal microbiome *in situ* as treatments for intestinal inflammation. For example, Hughes and colleagues found that molybdenum-cofactor-dependent metabolic pathways (such as nitrate reduction and formate oxidation) are a signature of inflammation-associated dysbiosis and contribute to the expansion of microorganisms, such as members of the Enterobacteriaceae family, which are dependent on such pathways.^246^ In subsequent work by the same group, authors were able to prevent expansion of Enterobacteriaceae during gut inflammation by tungsten treatment, which selectively inhibits molybdenum-cofactor-dependent microbial respiratory pathways.^247^ As such pathways are operational only during episodes of inflammation, tungsten treatment acts only on the enterobacterial population in the disease state, and does not affect Enterobacteriaceae during homeostatic conditions. This tungsten-mediated precision editing of the microbiota can therefore ameliorate the severity of intestinal inflammation in mouse models of colitis, and represents a promising and innovative avenue of research.

### Phage therapy: phages as a treatment vector

The use of phages as curative agents to treat bacterial infections has been applied for almost a century.^248–251^ However, due to variation in effectiveness and the discovery of antibiotics, the study of phages as therapeutics suffered a slow-down.^252^ More recently, with the rise of antibiotic resistance in bacteria, the need for alternative treatment solutions has re-focused the attention on phages. Phage therapy consists of administering lytic phages with the purpose of selectively removing specific bacteria that are thought to cause a disease, also known as “decolonization”.^253–256^ One of the main benefits of phage therapy is the ability to precisely target highly conserved species-specific membrane proteins and kill bacterial pathogens without disrupting the other members of the gut microbiota or attacking human cells.^254^ In addition, phage enzymes are able to disrupt bacterial biofilms, penetrating areas and infecting bacteria that are frequently difficult to reach by antibiotics.^257,258^ Some phage therapies have already been used in humans, including the use of phage preparations to treat *Pseudomonas aeruginosa* infections in burn wounds and chronic otitis,^251,254,259^ to treat gastrointestinal distress in donors with no diagnosed gastrointestinal condition,^260^ and as a last resource treatment in some cases of multidrug-resistant infections.^261–263^ Phage-based therapies are also being explored as treatment for UC and CD, and studies in mouse models of colitis have yielded encouraging results. In one study, mice infected with AIEC and administered with a cocktail of three AIEC-specific bacteriophages isolated from wastewater benefited from a significant decrease in fecal AIEC and reduced induced-colitis symptoms. In addition, the same phage cocktail was able to target AIEC in homogenates of ileal biopsies taken from CD patients.^264^ Similarly, another study identified a phage from the *Myoviridae* family, also isolated from wastewater that could reduce the abundance of diarrheagenic *E. coli* in a mouse model of intestinal colonization, while microbiome alpha and beta diversity remained unaltered.^265^ Efficacy of this phage’s *E. coli*-killing capabilities were also confirmed *in vitro*. Moreover, in a different study, three AIEC-specific *Caudovirales* phages isolated from an IBD patient reduced colonization of *E. coli* in germ-free mice.^164^ Using the same phage cocktail in a mouse model of colitis, however, authors observed that expansion in phage numbers exacerbated inflammation within the intestine and contributed to colitis symptoms, suggesting that, as seen in IBD, increased phage abundances may contribute to disease severity in chronic inflammation settings.

Despite some promising results obtained from animal models, the evidence of phage therapy efficacy on human IBD patients is still in its infancy. The safety and efficacy of EcoActive, a collection of bacteriophages against AEIC, is currently under investigation in a phase 2 double-blind, placebo-controlled trial with CD patients (www.clinicaltrials.gov: NCT03808103). Another phage cocktail therapy, BX002, is being evaluated as a treatment to target *Klebsiella pneumonia* in IBD/Primary Sclerosing Cholangitis (PSC) patients. The two diseases are thought to be related since a great proportion of PSC patients also suffer from IBD and previous studies have shown that *K. pneumonia* strains isolated from IBD/PSC patients are associated with the onset or exacerbation of both diseases in murine models.^266,267^ In a phase 1a single-blind, placebo-controlled trial, the safety, tolerability, and feasibility of delivering the phage cocktail BX002 to the recipients’ gut was demonstrated in healthy participants (NCT04737876). Based on these results, the efficacy of BX002 at reducing the levels of the target bacteria will be tested in a 1b/2a clinical trial. On the other hand, FMT could also represent an indirect tool of phage therapy, as previous work has shown transference of phages from donors to their recipients.^268,269^ In the study by Chehoud and colleagues, authors studied the viral fraction in stool samples after FMT from an adult donor to three pediatric patients with UC, and confirmed the transference of phages to the recipients.^269^ Similar results have been observed after FMT in the context of *Clostridium difficile* infections (CDI). Along with bacteria, viral populations were transferred from FMT donors to their recipients.^268^ Importantly, it has been suggested that the “phageome” alone may be sufficient to treat CDI. In a recent trial, the transference of fecal filtrates (devoid of whole bacteria but containing viral populations and bacterial products) from healthy donors to CDI patients with chronic relapse was able to restore normal stool habits and eliminated symptoms in recipients for at least six months.^270^ These studies support the theory that the effect of FMT could be mediated not exclusively by bacteria, and opens new phage applications to be explored to treat IBD and other intestinal diseases with microbial-dysbiosis components.

This approach, however, has some limitations. Extensive steps of purification of phage preparations are essential to eliminate bacterial residues, such as membrane components and endotoxins that may be present from propagating the phage in its host.^271^ The high specificity of phages is simultaneously an advantage and a limitation: while phages do not disturb microbial communities, and therefore are exempt of antibiotic-related side effects,^272^ target pathogens need to be identified beforehand, which narrows the spectrum of action and demands etiological understanding of the disease *a priori*.^254^ To avoid introducing harmful genes (i.e. antibiotic resistance or virulence factors) into the bacterial communities, only strictly lytic and fully sequenced phages should be utilized. However, lytic phage genomes contain many hypothetical genes with no known function. Additionally, the function and pathways of encoding proteins that alter bacterial physiology are not fully understood. As with antibiotic treatment, phage effectiveness can be hampered by bacterial resistance.^256^ Lytic phages exert strong antimicrobial selective pressures on their hosts, which possess a battery of anti-phage defense systems, including changes in receptor proteins that phages need to enter the cells, or CRISPR-Cas systems that recognize and degrade previously encountered foreign viral DNA.^273^ Simultaneously, phages continually evolve and adapt to maintain their infective capabilities, in an evolutionary race with their hosts.^256,274^ In the study by Goghokia and colleagues, *E. coli* strains developed resistance to phage therapy, but continuous administration of phage treatment was able to suppress bacterial growth.^164^ However, this process may not be sufficient to counter the emergence of phage-resistance when “mono-phage” therapies are being applied. Instead, the use of phage cocktails is a commonly used strategy that increases the spectrum of action.^254^ Nevertheless, use of such cocktails requires longer preparation and purification times, and have reduced pharmacokinetic predictability. Combination therapy of phages and lower concentration of antibiotics (enough to prevent cell division but not to cause cell death), known as “phage-antibiotic synergy” treatment has been suggested to be more effective against bacterial-associated diseases and at preventing the emergence of resistance.^254,275^ In addition, combining phage therapy with FMT may enhance microbiota engraftment and improve treatment efficacy. Overall, scientific evidence on phage therapy efficacy is still scarce, but there is a growing interest in the field and a strong need for novel therapeutics. Although some phage therapies are already in use in the food industry and agriculture, their use in humans has not been yet approved in the European Union or the United States.^256^ Adequate double-blind, randomized placebo-controlled trials are essential to prove their safety and efficacy to treat human infections and diseases, including IBD.

## Conclusions

In this review, we have provided an overview of recent technologies, both experimental and computational, used to analyze how the microbiome interacts with the host in IBD, and described their major advantages and limitations. Other technologies that have still not been extensively applied to the study of host–microbiome interactions in IBD include single-cell profiling and *in situ* characterization. Resolving heterogeneity of microbial species at the single cell level in complex communities is an important problem given the potentially large number of phenotypes arising from similar genotypes. Current approaches are limited to “bulk” estimates of composition and function, and approaches that can break down differences at higher resolution are necessary.^276^ Techniques for fluorescent hybridization or fluorescent spectral imaging can also be applied to visualize spatial structure of microbial communities, which can provide novel knowledge to understand community function and interaction with the host.^277,278^ There is also a pressing need to improve analytical techniques of microbiome data, including accurate approaches to reduce false correlations,^191^ methods to identify strain diversity,^279,280^ and characterize the increasing amount of genes and proteins of unknown function in microbial genomes.^281,282^

Finally, characterizing microbiome processes at the community level will be fundamental to better understand its role in IBD. Although many efforts are now directed toward characterization of specific isolates, consortia of microbes often interact in a synergistic manner to synthesize products that cannot be obtained in isolation.^283^ This is particularly relevant in IBD, as it has been previously demonstrated that the effect of SCFA to induce anti-inflammatory T_reg_s in the gut is not achievable by individual bacteria but require a defined set of bacteria.^284^ Further, microbes do not exist as isolated cells, but often form aggregates and biofilms, which are also highly relevant for IBD.^54^ Technologies that can better dissect such structures and can characterize bacterial signals at the community level are therefore necessary next steps to provide novel insights into IBD.
